# Perceived teacher-student relationship and growth mindset as predictors of student engagement in foreign student engagement in foreign language learning: the mediating role of foreign language enjoyment

**DOI:** 10.3389/fpsyg.2023.1177223

**Published:** 2023-05-22

**Authors:** Haoting Li

**Affiliations:** Marxist Institute, Ningbo University, Ningbo, China

**Keywords:** teacher-student relationship, growth mindset, learning enjoyment, student engagement, SEM

## Abstract

**Introduction:**

This study aimed to investigate the relationships between perceived teacher-student relationship, growth mindset, student engagement, and foreign language enjoyment (FLE) among Chinese English learners.

**Methods:**

A total of 413 Chinese EFL learners participated in the study and completed self-report measures for perceived teacher-student relationship, growth mindset, student engagement in foreign language learning, and FLE. Confirmatory factor analysis was employed to assess the validity of the scales. Structural equation modeling was used to test the hypothesized model.

**Results:**

The partial mediation model demonstrated the best fit to the data. The results indicated that perceived teacher-student relationship had a direct impact on student engagement. FLE directly influenced student engagement, while growth mindset indirectly affected student engagement through the mediation of FLE.

**Discussion:**

The findings suggest that fostering positive teacher-student relationships and promoting a growth mindset can enhance FLE, leading to increased levels of student engagement. These results emphasize the importance of considering both the interpersonal dynamics between teachers and students and the role of mindset in foreign language learning.

## Introduction

Learning a foreign language is a challenging task that requires considerable effort and dedication, requiring students’ considerable degrees of engagement (Tong et al., 2022). Student engagement is defined as a multidimensional learner-related construct that addresses students’ perceptual, sentimental, behavioral, and affective facets of learning (Reeve, 2013; Chang and Hall, 2022; Chiu, 2022). This concept has been discovered to be connected to students’ effective learning, preservation, tenacity, and learning perception (Dao and Sato, 2021; Hiver et al., 2021). On the other hand, the quality of teacher-student interactions in language classrooms is critical because second language (L2) instructors and students engage in interpersonal and interactional communication processes wherein instructors purposefully use effective interpersonal hints to inspire L2 learners and enhance the incidence of advantageous academic behaviors such as engagement (Mercer and Gkonou, 2020; Xie and Derakhshan, 2021; Derakhshan et al., 2022).

Positive teacher-student relationships have been shown to improve academic performance as well (Martin and Collie, 2019). Wubbels et al. (2016), and interplays between positive teacher-student relationships, academic performance, and accomplishment are prevalent (e.g., Wentzel and Wigfield, 1998; Valiente et al., 2008; Henry and Thorsen, 2018; Ma et al., 2018; Longobardi et al., 2021b; Kit et al., 2022; Sadoughi and Hejazi, 2022). When L2 learners and educators have a positive relationship, an effective classroom environment is created, students’ classroom enjoyment is enhanced, and students are more empathetically motivated to engage with the L2 teacher, curriculum content, activities, and the learning experience in general (Gabryś-Barker, 2016; Li, 2020; Mercer and Dörnyei, 2020). Positive interactions with educators can help students deal with the challenges of the classroom and encourage good learning habits (Longobardi et al., 2016; Henry and Thorsen, 2018). Generally, students who have trusting relationships with their instructors are more eager to learn, and they are more engaged in class activities (Martin and Dowson, 2009; Roorda et al., 2011; White, 2013; Claessens et al., 2017).

Moreover, individuals have various mindsets on the adaptability of human characteristics including personality, ethics, and intellectual ability (Dweck, 2006). Also, considering the inherent challenges of L2 learning, pupils’ opinions, mindsets, and perspectives on the adaptability of their competence have been shown to have an impact on their scholastic achievements (Dweck, 2017). In this sense, Dweck (1999) differentiates people’s mindsets into two categories: those who have a growth mindset and those with a fixed mindset. A growth mindset, also known as an incremental theory of intelligence, asserts that intellectual ability can be improved through training and strategy. People with a growth mindset maintain that by putting in more effort and observing difficulties as potential for advancement, they can improve their intelligence and cognitive operation (Papi et al., 2019; Derakhshan et al., 2022). Conversely, a fixed mindset, also known as the entity theory of intelligence, regards cognitive ability as a fixed and unalterable quality (Dweck, 2006). Students with a fixed mindset have a fixed view of their competence, maintaining that no amount of effort will modify their expertise (Lou and Noels, 2016; Zarrinabadi et al., 2022).

As another construct in this study, Foreign language enjoyment (FLE) is a notion that is compatible with the growing field of Positive Psychology, particularly the Broaden-and-Build Theory (Fredrickson, 2001). It promotes foreign language learning by encouraging learners to perform, invent, and discover a foreign linguistic and historical world (Li et al., 2018; Zeng, 2021). Enjoyment motivates students to discover the language, which improves their long-term persistence and durability (Dewaele et al., 2019). FLE, as opposed to negative feelings such as foreign language classroom anxiety (FLCA), seems to be more likely to be provoked by educators (Jiang and Dewaele, 2019) and has a greater impact on foreign language performance (Dewaele and Alfawzan, 2018).

Some studies have probed the teacher-student relationship, growth mindset, student engagement, and foreign language enjoyment separately (e.g., Hagenauer et al., 2015; Cavanagh et al., 2018; Martin and Collie, 2019; Tseng et al., 2020; Clem et al., 2021; Fathi and Mohammaddokht, 2021; Longobardi et al., 2021b; Hu et al., 2022; Kit et al., 2022; Xiao et al., 2022; Bai and Wang, 2023); however, to the best of our knowledge, no previous study has ever explored the simultaneous relationships between all of these variables in EFL contexts. Furthermore, previous studies have mainly focused on general education, rather than foreign language education or EFL contexts. This might be a notable gap in the literature, as foreign language education poses unique challenges and opportunities for both teachers and students (DiFino and Lombardino, 2004). Additionally, previous studies have primarily focused on direct effects, rather than indirect effects mediated by factors such as foreign language enjoyment.

As a result, having adopted Self-Determination Theory (SDT, Deci and Ryan, 1985), this study was set to investigate the role of perceived teacher-student relationship and growth mindset in predicting student engagement through the mediating role of FLE in the Chinese EFL context. Self-determination theory (Deci and Ryan, 1985), is one of the most influential theories in the field of motivation and has been widely used in educational contexts (Ryan and Deci, 2000). SDT emphasizes the significance of intrinsic motivation, which is driven by personal interests, values, and goals, and is associated with more positive learning outcomes than extrinsic motivation, which is driven by external rewards (Deci and Ryan, 1985; Ryan and Deci, 2000). The theory also proposes that autonomy, competence, and relatedness are essential needs that should be fulfilled to promote intrinsic motivation and wellbeing (Ryan and Deci, 2000). Investigating the relationship between these constructs, we can better understand the underlying mechanisms of foreign language learning and promote positive learning outcomes for EFL students. The findings may contribute to the existing literature by providing empirical evidence of the interplay among these factors and their effects on student engagement in learning a foreign language. Also, the novelty of this research lies in its examination of the mediating role of FLE in the relationship between growth mindset and student engagement, which has not been extensively studied in previous research. Additionally, this research focuses on the Chinese EFL context, which adds to the body of research on this topic in non-Western cultures.

## Literature review

### Student engagement

Scholars have extensively researched student engagement due to its significant impact on academic success or failure (Mystkowska-Wiertelak, 2022). However, L2 educators and researchers have only recently taken an interest in the concept (Derakhshan et al., 2022). Various definitions of student engagement exist, and in recent years, it has been conceptualized in different ways, including task-level engagement and overall engagement in a course (Reeve et al., 2020). Lamborn et al. (1992) described it as students’ psychological effort and investment in mastering course material, while Skinner et al. (2009) defined it as the quality of students’ participation and connection with the educational endeavor. In general, student engagement refers to students’ participation in classroom tasks, an indicator of motivation that fosters educational energy, investment, and progress (Skinner and Pitzer, 2012; Phillips, 2015). Student engagement is particularly vital in L2 education as engaged learners tend to be enthusiastic, dedicated, diligent, and determined (Hiver et al., 2021; Zhang, 2021).

Student engagement is a multifaceted construct encompassing behavioral, emotional, cognitive, and agentic dimensions (Henry and Thorsen, 2020; Derakhshan et al., 2022). Behavioral engagement pertains to students’ participation and active involvement in class discussions throughout a course, with predictors such as the quality and quantity of active participation (Philp and Duchesne, 2016; Phung, 2017; Fan and Xu, 2020). Cognitive engagement involves learners’ psychological investment in the learning process through the use of strategic tools (Li, 2021; Saeli and Cheng, 2021; Sun et al., 2021; Zhou et al., 2021). Emotional engagement focuses on students’ inner thoughts and feelings and how they respond to the learning experience, with emotionally engaged students being more enthusiastic, inclined to attend class, and having positive attitudes toward their education (McKellar et al., 2020). Lastly, agentic engagement pertains to students’ influence and role in enhancing learning and teaching efficiency (Reeve, 2013; Dao et al., 2021; Sun et al., 2021).

It’s important to note that these aspects of engagement can be influenced by various variables categorized by Guilloteaux (2016) as phenomenological (e.g., task complexity, potential, traditions, assignment, etc.), individual-demographic (e.g., age, gender, educational level, etc.), and instructional (e.g., teachers’ practices, behaviors, teaching method, ambition, ability, etc.) factors. Instructional and individual-demographic factors are often represented by teachers’ abilities to create a positive classroom social climate (CSC) and respond appropriately to students’ individual differences (IDs). Teachers who are skilled at fostering a positive CSC and addressing students’ mindsets can increase classroom engagement and reduce boredom in L2 classrooms (Derakhshan et al., 2022). Finally, researchers have concurred that student engagement is multidimensional, regardless of the fact that its scope and conceptualization are very divergent. They contend that a variety of elements, including student academic engagement, contribute to students’ positive attitudes toward the learning process (Fredricks et al., 2004; Reeve and Tseng, 2011; Carter et al., 2012; Phan, 2014; Lei et al., 2018; Zhao et al., 2021).

### Teacher-student relationship and student engagement

The concept of rapport is closely linked to caring, as it is a relational aspect that consistently results in positive educational outcomes (Frisby, 2019). Rapport is defined as warm and likable interactions between individuals, as well as a trusting relationship based on numerous positive interpersonal exchanges (Frisby and Housley Gaffney, 2015; Meng, 2021). Frisby and Martin (2010) describe rapport as an overall sentiment between two individuals, comprising a mutual, trustworthy, and prosocial bond. Teachers can establish a robust rapport with their students by demonstrating respect for their opinions and addressing their educational requirements, as Thompson (2018) suggests. del Carmen Santana (2019) proposed that positive teacher-student relationships can be established by recognizing students’ efforts and providing positive feedback. Students generally consider the teacher-student relationship to be a fundamental component of effective instruction (Burke-Smalley, 2018; Martin and Collie, 2019; Clem et al., 2021; Longobardi et al., 2021a,b).

Positive relationships in the classroom foster a favorable learning environment, positive classroom experiences, positive learning attitudes, and improved performance (Delos Reyes and Torio, 2021; Song et al., 2022). Instructors can elicit a feeling of intimacy and kindness among students by engaging in a variety of nonverbal communication behaviors (Burke-Smalley, 2018). Such behaviors include caring for students, honoring their viewpoints, permitting learners to openly share their thoughts, being friendly, accessible, and providing appropriate feedback (Burke-Smalley, 2018). Due to the distinctive social and interpersonal nature of L2 classrooms, developing teacher-student rapport is a crucial component of successful L2 learning and teaching (Sánchez et al., 2013). Effective teacher-student relationships are positively correlated with academic expertise and success (Wubbels et al., 2016; Longobardi et al., 2021b), and these relationships have favorable impacts on educational results (e.g., Valiente et al., 2008; Henry and Thorsen, 2018). Favorable relationships with educators can help students deal with the challenges of the classroom and promote positive learning habits (Roorda et al., 2011). Students who have trusting relationships with their instructors are more inclined to learn, and they are more engaged in class (Martin and Dowson, 2009; Wentzel, 2009; White, 2013; Claessens et al., 2017). Generally, pupils are more empathetically encouraged to engage with the L2 teacher, course, activities, and the learning process if a positive relationship is found between L2 students and the instructor. This results in a positive classroom climate, classroom enjoyment, and a positive classroom climate (Gabryś-Barker, 2016; Li, 2020 Mercer and Dörnyei, 2020; Kit et al., 2022).

Numerous research studies have investigated the impacts of the student-teacher relationship on learners and their academic behaviors. According to Yong (2019), close teacher-student relationships can lead to improved learning outcomes, as strong bonds between teachers and students motivate students to invest more time in their academic work (Rowan and Grootenboer, 2017). Positive interactions between teachers and students can also play a key role in motivating behavior in the classroom and significantly increase students’ levels of motivation (Meng, 2021). A study by Culpeper and Kan (2020) found that improving teacher-student relationships correlated with increased engagement in online classes among university L2 learners, while Wilson et al. (2010) discovered that rapport can predict students’ inspiration and level of engagement in the classroom. Similarly, Thornberg et al. (2022) found that positive teacher-student relationships can foster students’ engagement in the classroom. Cai (2021) also found that a close teacher-student relationship positively predicted L2 students’ willingness to communicate. The quality of teacher-student interactions is essential for comprehending student engagement, and individualized feedback and guidance can improve student engagement (Pianta et al., 2012). Higher quality interactions lead to more favorable school attitudes, more engagement, improved math and reading performance, increased intimacy with instructors, and decreased conflict with instructors among students (LoCasale-Crouch et al., 2018). Ryan and Patrick (2001) found that all major changes in motivation and engagement were attributed to a higher-order social environment element in the classroom, and positive changes were associated with teacher support, facilitating interaction, and mutual respect.

### Growth mindset

Dweck (2006) proposed that individuals possess implicit theories regarding various traits, such as talent, intelligence, character, and management, which may take the form of either fixed or growth mindsets. A growth mindset is concerned with the belief that one’s language ability can improve through effort, appropriate strategies, and work, while a fixed language mindset implies that language aptitude is immutable (Lou and Noels, 2016). According to Mercer and Ryan (2010), learners have both fixed and growth mindsets about their language proficiency, influenced by their language background and social factors. An individual’s mindset pertains to their perception of their ability to cope with obstacles (Dweck, 2017). Learning a second language is considered challenging due to its multifaceted nature, which compels learners to venture beyond their comfort zones (Cacali, 2019; Hu et al., 2022). Their language attitudes determine their ability to handle and overcome difficulties. In general, learners’ mindsets and beliefs regarding their capabilities and academic accomplishments play a crucial role in the L2 learning process (Cacali, 2019). Furthermore, these beliefs affect how students interpret failure and cope with the many setbacks encountered during L2 instruction (Dweck, 2006). Those with a fixed mindset often view their academic success, struggles, and other factors as reflections of their intelligence and competence (Zeng et al., 2016). Conversely, individuals with a growth mindset typically perceive their academic lives as opportunities for learning, growth, and development. They regard obstacles, difficulties, and efforts as productive means of enhancing their knowledge, skills, and experiences (Zeng et al., 2016).

Studies suggest that students who have a growth mindset tend to adopt more learning objectives and respond to failure situations in a more mastery-oriented manner, while those with a fixed language attitude tend to embrace performance-based goals and exhibit helpless-oriented behavior when they fail (Lou and Noels, 2016; Cavanagh et al., 2018; Wang and Amemiya, 2019; Bostwick et al., 2020; Tseng et al., 2020). Previous research has investigated the link between language mindsets and emotions, and it has been found that students with a fixed language mindset often report experiencing more stress during their social and interpersonal interactions due to concerns about being ignored by those who speak the target language due to their low proficiency. In contrast, students with a growth language mindset show greater adaptability when faced with a novel sociocultural setting (Lou and Noels, 2019; Khajavy et al., 2022). In addition, previous studies have demonstrated that a growth language mindset affects L2 speaking self-confidence, while a fixed language mindset is a strong correlate of L2 speaking anxiety (Ozdemir and Papi, 2022).

Recent research has also explored the interconnection between language mindsets and other psychological and affective factors. For example, Lou and Noels (2017) found that growth mindsets positively influenced learning objectives and reactions, while fixed mindsets were negatively correlated with educational objectives. Moreover, according to Bai et al. (2021), growth mindsets were interconnected with higher levels of self-efficacy, writing motivation, and self-monitoring. Similarly, Waller and Papi (2017) found that growth mindsets were a good predictor of writing motivation. However, Khajavy et al. (2021) found that although growth mindset was a minor predictor of students’ achievement, it was still a strong one. Furthermore, Eren and Rakıcıoğlu-Söylemez (2020) conducted a study with 526 EFL students and discovered strong and partial interconnections between language mindsets, four dimensions of engagement, perceived instrumentality, and graded performance.

### Foreign language enjoyment

The experience of enjoyment has been one of the trendiest subjects in empirical investigations on foreign language instruction, which resonates with the introduction of positive psychology (PP) in second language acquisition (SLA) (Li et al., 2018; Guo, 2021; Zeng, 2021). Researchers’ interest in SLA’s enjoyment grew out of their investigation into a potential connection between this pleasant feeling and academic achievement in L2 learners. Dewaele and MacIntyre (2014) achieved a significant advancement in the study of enjoyment by drawing on the insights of PP. They established the Foreign Language Enjoyment Scale, the primary tool used to measure FLE, based on Likert scale evaluations of 21 items, and formally presented the idea of FLE in SLA. This study examined the connection between FLE and FLCA using an online survey. The statistical analysis demonstrated that FLE and FLCA were two distinct dimensions rather than two opposite sides of the same coin; learners’ FLE levels were considerably higher than their FLCA levels, and there was a slight negative relationship between the two experiences (Dewaele and MacIntyre, 2014). Additionally, they were the first investigators to make the assertion that both feelings differ according to gender, with female volunteers apparently feeling more FLE and FLCA, compared to men (Guo, 2021). Dewaele and MacIntyre (2016) differentiated between enjoyment and pleasure based on the respondents’ descriptions of their most pleasurable experiences and conceived enjoyment as a “complex emotion, capturing interacting dimensions of challenge and perceived ability that reflect the human drive for success in the face of difficult tasks” (Dewaele and MacIntyre, 2016, p. 217).

Furthermore, they contended that FLE was mediated by both private factors like students’ feelings of fulfillment and achievement, as well as social factors like a positive environment with a good teacher and encouraging peers. Dewaele et al. (2016) used data from the FLE Scale and an open question to investigate the difference between men and women in FLE and FLCA. The results showed that female participants considerably enjoyed more and had less FLCA in the FL classroom, correlating with the outcomes of the prior research (Dewaele and MacIntyre, 2014). Piechurska-Kuciel (2017) examined the connection between language proficiency and enjoyment among learners. The findings showed that while strong social connections with teachers and peers fostered FLE, language proficiency was also found to be favorably related to this feeling of wellbeing. This happens because greater language proficiency is typically associated with a higher sense of control, and competent learners are more likely to benefit from an appreciation of the importance of language ability. Elahi Shirvan and Talebzadeh (2017) utilized an idiodynamic approach to record the rapid moment-to-moment fluctuations of this pleasurable experience in order to analyze the impact of various conversational subjects on the dynamics of FLE. According to the study’s findings, FLE is a dynamic system that varies on an interpersonal and intrapersonal level, and the topic is an attractor for students’ enjoyment. These findings suggest that teachers can increase the enjoyment of language learners by selecting appropriate conversational topics and managing the level of entertainment and difficulty in classroom discussions. Dewaele et al. (2018) examined the nature and strength of relationships between learner-internal and teacher-specific variables and FLE and FLCA. The findings revealed that both learner-internal and classroom-specific factors affected FLE and FLCA. To clarify, pupils with more experience and skill had greater levels of FLE. Furthermore, it was noted that higher levels of FLE were correlated with student enthusiasm for the FL and the FL teacher, the teacher using the FL extensively in class, and the amount of time students engaged in speaking. The enhancement of students’ FLE was found to be more significantly influenced by instructors than the reduction of their FLCA (Guo, 2021).

De Smet et al. (2018) investigated how FL learners’ FLE and FLCA were impacted by the target language. They discovered that, compared to monolinguals, bilinguals demonstrated higher levels of FLE and lower levels of FLCA. The mechanisms and characteristics of enjoyment transmission in a foreign language course were investigated by Talebzadeh et al. (2020). The results showed that the primary mechanism of enjoyment transmission in teacher-student interactions was automatic mimicking. This was also influenced by the use of face movements, body language, and postures, including smiling, nodding, and leaning forward.

In a study done by Dincer et al. (2019), it was concluded that the degree to which students perceived their instructors’ support for their autonomy in the classroom influenced their satisfaction of need, which in turn increases self-determined engagement. Engagement in English courses was a strong predictor of success and attendance. Also, semi-structured interviews revealed findings that were in line with the quantitative findings, and learners also expressed the opinion that their engagement would be best promoted in classes with a friendly social environment. Additionally, their responses emphasized how crucial language teachers are in assisting students in meeting their psychological needs, engaging in class, and achieving academic success. Furthermore, Dewaele et al. (2022b) conducted a study to investigate the foreign language enjoyment (FLE), foreign language classroom anxiety (FLCA), and attitude/motivation (AM) of 360 students as influenced by three teacher behaviors. The results of this study revealed a positive relationship between the three teacher behaviors, FLE, and AM, but no significant relationship with FLCA. Moreover, Dewaele et al. (2022a) concluded that FLE can significantly increase learners’ motivation, which in turn increases students’ engagement.

## The present study

This research is based on the theoretical framework of self-determination theory (Deci and Ryan, 1985), which suggests that students’ engagement and motivation are influenced by the degree to which their needs for autonomy, competence, and relatedness are met. Autonomy refers to the extent to which students are able to make choices and exert control over their learning. Competence refers to the sense of efficacy and mastery that students have in relation to their learning tasks. Relatedness refers to the social connectedness and sense of belongingness that students experience in their learning environment. Perceived teacher-student relationship is considered an important factor that contributes to the satisfaction of these needs (Ryan and Deci, 2000). Additionally, growth mindset has been found to be associated with greater autonomy, competence, and relatedness (Yeager and Dweck, 2012). Finally, FLE has been found to be associated with greater autonomy, competence, and relatedness, as well as greater degrees of motivation and achievement (Dewaele and MacIntyre, 2016).

Therefore, the following hypotheses were formulated:

H1: Perceived teacher-student relationship affects FLE directly.

Theoretical models of motivation and engagement suggest that the relationship between teachers and students can impact students’ engagement through affective variables such as emotions, attitudes, and motivation (Wentzel, 2009; Culpeper and Kan, 2020; Meng, 2021; Thornberg et al., 2022). According to SDT (Ryan and Deci, 2000), students’ need for relatedness is one of the three basic psychological needs that should be satisfied in order to foster intrinsic motivation and engagement in the classroom (Deci and Ryan, 1985). In addition, social cognitive theory emphasizes the importance of social relationships in shaping individuals’ beliefs, attitudes, and behaviors (Bandura, 1989). Thus, seems logical to hypothesize that the quality of the teacher-student relationship would have a direct impact on students’ FLE. Specifically, a positive and supportive teacher-student relationship would provide learner with a sense of belonging, autonomy, and competence, which are all important factors that influence students’ FLE (Dewaele et al., 2019). As such, H1 is theoretically warranted.

H2: Students’ growth mindset affects FLE directly.

According to SDT, students’ need for competence is another important psychological need that should be satisfied in order to foster intrinsic motivation and engagement in the classroom (Deci and Ryan, 1985). Growth mindset can help students develop a sense of competence by promoting a focus on effort and learning (Dweck, 2006, 2017). Moreover, growth mindset can help students overcome obstacles and persist in the face of challenges, which are important factors that influence students’ FLE (Hu et al., 2022).

H3: FLE influences student engagement directly.

The broaden-and-build theory of positive emotions suggests that positive emotions, such as enjoyment, broaden individuals’ cognitive and behavioral repertoires, which can lead to increased learning and academic achievement (Fredrickson, 2001). FLE can be considered as a positive emotion that arises from the learning experience and enhances students’ motivation and engagement in the classroom (Dewaele and MacIntyre, 2014). FLE can also lead to a positive attitude toward language learning, which can further enhance students’ engagement (Guo, 2021; Mohammad Hosseini et al., 2022).

H4: FLE mediates the link between perceived teacher-student relationship and student engagement.

According to the tripartite model of engagement, engagement is a multifaceted construct that includes behavioral, emotional, and cognitive components (Skinner and Belmont, 1993). FLE can be considered as an emotional component of engagement that is closely related to students’ affective experiences in the classroom. Positive emotions, such as enjoyment, can promote students’ motivation and persistence in learning, which in turn can enhance their engagement with the learning material (Fredrickson, 2001; Frisby, 2019). Also, perceived teacher-student relationship has been found to have a significant impact on students’ emotional experiences in the classroom (Martin and Dowson, 2009; Wentzel, 2009; Wilson et al., 2010; Thornberg et al., 2022). Specifically, a positive and supportive teacher-student relationship can create a sense of trust, safety, and belongingness that enhances students’ emotional wellbeing and positive affective experiences in the classroom (Roorda et al., 2011). As a result, it is hypothesized that FLE mediates the link between teacher-student relationship and student engagement, since FLE can act as a mechanism through which the emotional impact of the teacher-student relationship is translated into students’ engagement with the learning material (Dewaele et al., 2022a).

H5: FLE mediates the link between growth mindset and student engagement.

Growth mindset is a psychological construct that refers to the belief that one’s abilities can be developed through hard work, effective strategies, and feedback (Dweck, 2006). Students with a growth mindset tend to have a more positive and adaptive approach to learning, which can enhance their engagement with the learning material (Yeager and Dweck, 2012). In addition, FLE might be positively associated with growth mindset, as students who enjoy learning are more likely to believe that their efforts will lead to improvement (Bai and Wang, 2023). If students with a growth mindset are more likely to enjoy learning, and FLE is a significant predictor of student engagement, then it follows that FLE can serve as a mechanism through which growth mindset influences student engagement. In other words, students with a growth mindset may be more engaged with the learning material because they enjoy learning, and this enjoyment may be fostered by the belief that their efforts will lead to improvement (Zhao et al., 2018).

Based on the hypotheses, the research questions for this study are:

1. Does perceived teacher-student relationship impact FLE?
2. Does students’ growth mindset impact FLE?
3. Does FLE affect student engagement?
4. Does FLE mediate the relationship between perceived teacher-student relationship and student engagement?
5. Does FLE mediate the relationship between growth mindset and student engagement?

## Materials and methods

### Participants

To accomplish the purpose of this survey, a number of 413 Chinese EFL learners (225 females and 188 males) were recruited as the participants. They had an age range of 18–25 years (*M* = 20.56, SD = 1.73) from different universities in China. Specifically, they were recruited from three universities in eastern China: Ningbo University in Ningbo, Zhejiang University in Hangzhou, and Suzhou University in Suzhou. The students were all enrolled in English language courses at intermediate or advanced proficiency levels, and had been learning English for at least 6 years. The intermediate and advanced proficiency levels were determined based on the students’ English language proficiency scores on the Test for English Majors (TEM) administered by the Chinese Ministry of Education. Students who scored between 70 and 79 on the TEM were classified as intermediate level, and those who scored 80 and above were classified as advanced level. The TEM is a widely recognized proficiency test for English majors in China and is administered to students in their third or fourth year of university study. The participants were selected through convenience sampling. The demographic information of the participants is illustrated in Table 1.

**TABLE 1:** Participant demographics.

Variable	*N*	Percentage
**Gender**		
Female	225	54.5%
Male	188	45.5%
Age (years)	413	
Mean (SD)	20.56	1.73
**Proficiency**		
Intermediate	163	39.5%
Advanced	250	60.5%
**University**		
Ningbo University	163	39.5%
Zhejiang University	125	30.3%
Suzhou University	125	30.3%
Total	413	100%

## Instruments

### Perceived teacher-student relationship scale

To assess the quality of the relationship between teachers and students, a questionnaire was used, originally created by Pianta and Nimetz (1991) and modified by Wang and Wang (2002) to suit Chinese culture. The questionnaire consists of 15 items and evaluates the students’ perception of three aspects of their relationship with their teacher: closeness, positive reactivity, and conflict. Participants assessed the extent to which each statement applied to their current head teacher on a scale of 1 (definitely does not apply) to 5 (definitely applies). The scores were obtained by averaging the responses to the items, with a higher score indicating a more positive teacher-student relationship.

### Growth mindset scale

The growth mindset of the participants was evaluated using a scale created by Papi et al. (2019), which was originally derived from Dweck’s (2006) mindset scale. The self-report questionnaire includes four items, each rated on a 6-point Likert scale ranging from 1 (“strongly disagree”) to 6 (“strongly agree”). An example statement from the scale is “I can change even my basic language learning intelligence considerably.”

### Student engagement scale

To assess the level of involvement of the participants in the research, the Student Engagement Scale was employed. The scale was verified by Reeve (2013) and was intended for use with university students. The scale assesses four elements of the construct which are Agentic Engagement (5 items), Behavioral Engagement (4 items), Cognitive Engagement (4 items), and Emotional Engagement (4 items). Each item in the scale is assessed on a 7-point Likert scale ranging from 1 (strongly disagree) to 7 (strongly agree). Reeve (2013) attested to the good reliability of all four dimensions of the scale.

### Foreign language enjoyment scale

In order to evaluate the level of satisfaction of EFL students, the measurement tool created by Jiang and Dewaele (2019) was utilized. The measurement tool consists of ten statements which were taken from the original scale that was confirmed by Dewaele and MacIntyre (2014). The scale examines both personal and social aspects of enjoyment. The reliability of this scale was found to be high by Jiang and Dewaele (2019), with a coefficient of α = 0.88. An example item from the scale is “It is cool to know English as a foreign language.”

### Procedure

Participants were recruited from three universities in China. Inclusion criteria for participants were that they were Chinese EFL learners who had been learning English for at least 3 years, and were currently enrolled in an English course. Participants were recruited through convenience sampling methods, and those who met the inclusion criteria were invited to participate in the study. Prior to collecting the data, the researcher obtained ethical clearance from the Institutional Review Board (IRB) of Ningbo University. The IRB reviewed and approved the study protocol, including the study design, data collection procedures, and measures. Participants were provided with a consent form that explained the purpose of the study, assured confidentiality and anonymity of their responses, and allowed them to withdraw from the study at any time. The self-report questionnaire that contained four measures: perceived teacher-student relationship, growth mindset, student engagement, and FLE was distributed to participants during their English classes, and they were instructed to complete it in their own time. Upon completion, the questionnaires were collected by the researcher and entered into a database for analysis.

### Data analysis

The data collected were first analyzed descriptively using SPSS version 23.0. Correlation analyses were performed to examined the correlations among the variables of interest. To test the research hypothesis, Structural Equation Modeling (SEM) was performed using the Amos program (version 22.0). The measurement model was fitted to the data first, following the two-step approach recommended by Anderson and Gerbing (1988). In this step, the construct validity of the measurement model was assessed by examining the factor loadings, composite reliability, and average variance extracted (AVE) of the latent variables. Next, the structural model was examined to test the relationships among the latent variables. Baron and Kenny’s (1986) method was used to examine the mediation analysis. The fit of the model was evaluated using several fit indices. The χ^2^/df ratio, with a *p*-value greater than 0.05, was used to assess the goodness-of-fit of the model (Chau, 1997). Additional fit indices, including the Goodness of Fit Index (GFI) and the Comparative Fit Index (CFI), were considered good if they had values of 0.90 or higher (Chau, 1997). The Root-Mean-Square Error of Approximation (RMSEA) and the Standardized Root-Mean-Square Residual (SRMR) were also employed to assess model fit, with values of RMSEA < 0.08 and SRMR < 0.10 considered indicative of good fit (Vandenberg and Lance, 2000).

Data Analysis The data collected were first analyzed descriptively using SPSS version 23.0. Correlation analyses were performed to examine the relationships among the variables of interest. To test the research hypothesis, Structural Equation Modeling (SEM) was performed using the Amos program (version 22.0) to estimate the structural equation model. The measurement model was fitted to the data first, following the two-step approach recommended by Anderson and Gerbing (1988). In this step, the construct validity of the measurement model was assessed by examining the factor loadings, composite reliability, and average variance extracted (AVE) of the latent variables. To test for the mediation effect, the Sobel test was used with bootstrapping and confidence intervals, as recommended by the reviewer. Next, the structural model was examined to test the relationships among the latent variables. The fit of the model was evaluated using several fit indices. The χ^2^/df ratio, with a *p*-value greater than 0.05, was used to assess the goodness-of-fit of the model (Chau, 1997). Additional fit indices, including the Goodness of Fit Index (GFI) and the Comparative Fit Index (CFI), were considered good if they had values of 0.90 or higher (Chau, 1997). The Root-Mean-Square Error of Approximation (RMSEA) and the Standardized Root-Mean-Square Residual (SRMR) were also employed to assess model fit, with values of RMSEA < 0.08 and SRMR < 0.10 considered indicative of good fit (Vandenberg and Lance, 2000).

## Results

Table 2 shows the means, standard deviations, and correlations among the constructs. All variables were significantly correlated with each other in the expected direction. Specifically, perceived teacher-student relationship was positively correlated with growth mindset (*r* = 0.34, *p* < 0.001), student engagement (*r* = 0.43, *p* < 0.001), and FLE (*r* = 0.30, *p* < 0.001). Growth mindset was positively correlated with student engagement (*r* = 0.50, *p* < 0.001) and FLE (*r* = 0.47, *p* < 0.001). Student engagement was also positively correlated with FLE (*r* = 0.56, *p* < 0.001).

**TABLE 2:** Descriptive statistics.

	Mean	*SD*	1	2	3	4
1. Teacher-student relationship	3.51	0.71	-			
2. Growth mindset	3.71	0.66	0.34**	–		
3. Student engagement	3.76	0.69	0.43**	0.50**	–	
4. Foreign language enjoyment	3.59	0.70	0.30**	0.47**	0.56**	-

**p < 0.01.

Before testing the structural model, the measurement model was tested to assess the validity and reliability of the measurement instruments. Confirmatory factor analysis was conducted using AMOS 22.0. The measurement model included four latent variables (perceived teacher-student relationship, growth mindset, student engagement, and FLE) with four observed indicators for each variable. The fit indices for the measurement model were satisfactory: χ^2^ (148) = 312.08, *p* < 0.001, CFI = 0.96, TLI = 0.95, RMSEA = 0.05, 90% CI [0.04, 0.06], SRMR = 0.03. As seen in Table 3, all factor loadings were significant (*p* < 0.001) and ranged from 0.64 to 0.91, indicating good convergent validity. The average variance extracted (AVE) was calculated for each latent variable, which indicates the amount of variance captured by the indicators of the latent variable. The AVEs were 0.69 for perceived teacher-student relationship, 0.74 for growth mindset, 0.68 for student engagement, and 0.75 for FLE, all above the recommended threshold of 0.50 (Fornell and Larcker, 1981), indicating good convergent validity. Cronbach’s alpha coefficients were calculated for each latent variable to assess internal consistency reliability, and they ranged from 0.81 to 0.90, indicating good reliability.

**TABLE 3:** Measurement model results.

Latent variable	Indicator 1	Indicator 2	Indicator 3	Indicator 4	Factor loading	AVE	Cronbach’s alpha
Teacher-student	Ptr1	Ptr2	Ptr3	Ptr4	0.86	0.69	0.87
Growth mindset	Gm1	Gm2	Gm3	Gm4	0.91	0.74	0.90
Student engagement	Se1	Se2	Se3	Se4	0.64	0.68	0.81
FLE	FLE1	FLE2	FLE3	FLE4	0.85	0.75	0.84

Ptr, perceived teacher-student relationship; Gm, growth mindset; Se, student engagement; FLE, foreign language enjoyment; AVE, average variance extracted. All factor loadings were significant at *p* < 0.001.

Also, additional analyses were carried out to examine the fit of the hypothesized model compared to higher or lower-order models. Specifically, the proposed model was compared with a higher-order model that included a latent variable representing general academic achievement and a lower-order model that excluded the latent variable of perceived teacher-student relationship. Results of these analyses (see Table 4) revealed that the proposed model with perceived teacher-student relationship and growth mindset as predictors of student engagement with the mediating role of FLE had a significantly better fit than the higher-order model (Δχ^2^ = 25.30, Δdf = 3, *p* < 0.001) and the lower-order model (Δχ^2^ = 16.82, Δdf = 2, *p* < 0.001).

**TABLE 4:** Model comparison.

Model fit indices	Proposed model	Higher-order model	Lower-order model
χ^2^	130.50	155.80	147.32
Df	89	92	91
χ^2^/df	1.46	1.69	1.62
CFI	0.94	0.91	0.92
TLI	0.92	0.88	0.89
RMSEA	0.05	0.07	0.06
SRMR	0.04	0.06	0.05
Δχ^2^ vs. higher-order model	25.30*	–	–
Δdf vs. higher-order model	3	–	–
*p*-value vs. higher-order model	<0.001	–	–
Δχ^2^ vs. lower-order model	16.82*	–	–
Δdf vs. lower-order model	2	–	–
*p*-value vs. lower-order model	<0.001	–	–

*Indicates a significant difference at *p* < 0.05. χ^2^, chi-square; df, degrees of freedom; CFI, Comparative Fit Index; TLI, Tucker-Lewis Index; RMSEA, Root Mean Square Error of Approximation; SRMR, Standardized Root Mean Square Residual.

After validating the measurement model, alternative structural models were examined to test the hypotheses. Specifically, the hypothesized partial mediation model (Model 1) was compared with a full mediation model (Model 2) and a direct model (Model 3). The fit statistics of all three models are shown in Table 5. The hypothesized model (Model 1) had a significantly better fit than Model 2 and model 3, based on the used fit indices. Thus, Model 1 was considered as the most parsimonious fit to the data.

**TABLE 5:** Fit indices of the direct effect, full mediation, and partial mediation models.

Model	χ^2^	Df	*P*-value	CFI	TLI	RMSEA	SRMR
Direct effect model (1)	300.16	146	<0.001	0.95	0.93	0.06	0.04
Full mediation model (2)	262.41	145	<0.001	0.96	0.95	0.05	0.03
Partial mediation model (3)	187.91	144	<0.001	0.98	0.96	0.04	0.02

**FIGURE 1:**
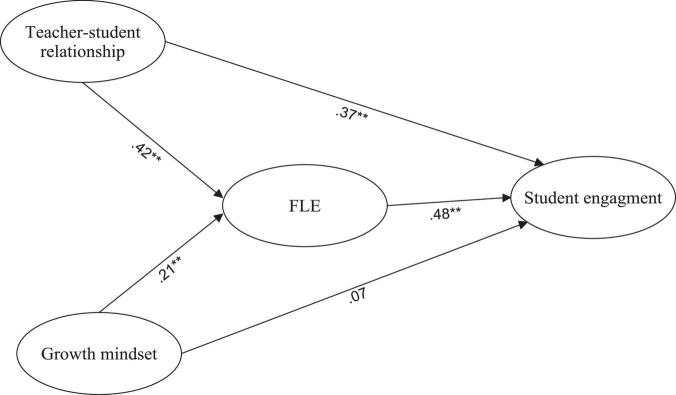
Final fit model.

Figure 1 shows the path and parameter estimates for the final fit model (Model 1). As seen in the Figure 1, all the path coefficients were significant except for the path between growth mindset and student engagement. The structural model indicated that teacher-student relationship significantly influenced FLE (β = 0.42 *p* < 0.01). Similarly, growth mindset had a significant positive effect on FLE (β = 0.21, *p* < 0.01). Additionally, FLE was positively related with student engagement (β = 0.48, *p* < 0.01).

Afterward, Baron and Kenny’s (1986) method was used to test whether FLE mediated the relationship among variables. The direct model (Table 6) revealed significant path coefficients between teacher-student relationship, growth mindset, and student engagement (teacher-student relationship→ student engagement: 0.39, *p* < 0.001; growth mindset→ student engagement: 0.16, *p* < 0.05), which confirms the first step of Baron and Kenny’s method. The full mediation model found significant path coefficients between teacher-student relationship and growth mindset on FLE (teacher-student relationship→ FLE: 0.44, *p* < 0.001; growth mindset→ FLE: 0.23, *p* < 0.01), which confirmed the second step of the method. The partial mediation model showed that FLE partially mediated the relationship between growth mindset and student engagement. In addition, growth mindset had an insignificant path coefficient on student engagement, while FLE was a full mediator between teacher-student relationship and student engagement. Thus, the influence of growth mindset on FLE affected student engagement.

**TABLE 6:** Path estimates of structural model.

Standardized path coefficients (*t*-value)			
	**Direct effects model**	**Full mediation model**	**Partial mediation model**
TSR → ENG	0.39 (4.98***)		0.37 (4.12***)
GROW → ENG	0.16 (2.89*)		0.07 (0.62)
TSR → FLE		0.44 (4.12***)	0.42 (3.95***)
GROW → FLE		0.23 (3.47**)	0.21 (3.02**)
FLE → ENG		0.51 (8.85***)	0.48 (8.12***)

TSR, teacher-student relationship; GROW, growth mindset; ENG, student engagement; FLE, foreign language enjoyment. *p-value < 0.05, **p-value < 0.01, ***p-value < 0.001.

## Discussion

The findings obtained from the data analysis of this study revealed several important findings that have implications for foreign language education and the development of effective strategies to support student engagement and motivation in foreign language learning. Firstly, the results of the study showed that perceived teacher-student relationships had a direct impact on student engagement of Chinese EFL learners. This finding agrees with previous research that has underscored the significance of positive relationships between teachers and students for promoting student motivation, engagement, and wellbeing in the classroom (Martin and Dowson, 2009; Wentzel, 2009; Wilson et al., 2010; White, 2013; Claessens et al., 2017; Culpeper and Kan, 2020; Meng, 2021; Thornberg et al., 2022). The finding supports the results of Thornberg et al. (2022), who concluded that the level of student engagement in the classroom can be considerably increased by a positive teacher-student rapport. This finding highlights the importance of fostering positive relationships between instructors and pupils in foreign language classrooms, which may help to enhance students’ engagement and ultimately their language learning outcomes. Teachers who establish positive and supportive relationships with their students can create a supportive and inclusive learning environment, which can help students feel more connected to their teachers, peers, and the subject matter and ultimately enhance their engagement in the foreign language learning process. According to the findings of the current study, greater feelings of rapport and engagement occur simultaneously (Culpeper and Kan, 2020). This conclusion can be theoretically explained by referring to the relative nature of language instruction (Mercer and Dörnyei, 2020), which encourages language teachers to use positive (nonverbal) communication cues, develop teacher-student rapport, and convey positive emotions and sensations since, in addition to promoting student engagement, good teacher feelings and interpersonal behaviors are major accelerators of any favorable L2 learning experience (Hiver et al., 2021). Moreover, consistent with the findings of the current study, Yong (2019) asserted that strong teacher-student relationships will eventually lead to enhanced academic achievement. This is mostly due to the close relationships that teachers develop with their pupils, which inspire them to put greater effort into their academic work.

The second finding was that FLE directly affected student engagement of EFL learners. The results of the study also showed that FLE had a direct impact on student engagement. This supports prior research that has found that students’ enjoyment of a subject can be a powerful predictor of their motivation and engagement in learning (Dewaele et al., 2018). By promoting an enjoyable and engaging learning experience, teachers can increase students’ intrinsic motivation and, as a result, improve their engagement in foreign language learning. In line with this finding, in Dincer et al.’s (2019) study, students indicated that classes with a welcoming social atmosphere would best foster their engagement. Partially in line with this finding, Dewaele et al. (2022b) mentioned a positive correlation between teacher behaviors, FLE, and Attitude/Motivation, but no significant correlation with FLCA. Furthermore, Dewaele et al. (2022a) argued that high FLE can become intrinsically motivating by acting as a shield to improve low motivation.

Finally, the results of the study showed that a growth mindset had an indirect impact on student engagement through the mediation of FLE in the EFL context. This finding suggests that students who possess a growth mindset, or the belief that their abilities and intelligence can be developed through effort, are more likely to experience higher levels of foreign language enjoyment, which, in turn, enhances their engagement in foreign language learning. This finding is in line with previous research that has demonstrated the importance of growth mindset in promoting academic achievement and motivation (e.g., Dweck, 2006). The results suggest that promoting a growth mindset among Chinese English learners may be an effective strategy to enhance their FLE and engagement in learning English. Teachers can encourage a growth mindset by emphasizing the importance of effort and persistence, praising students’ progress rather than their innate ability, and providing opportunities for students to learn from their mistakes (Blackwell et al., 2007). This finding is consistent with previous research that has shown a positive relationship between a growth mindset and student motivation and engagement in academic domains (Dweck, 2006; Eren and Rakıcıoğlu-Söylemez, 2020; Khajavy et al., 2021). The results also showed that learners with a growth mindset believed that their abilities and intelligence are adaptable and can be increased with more effort; they persevered in the face of L2 learning difficulties and showed greater self-control, enjoyment, and engagement. In line with this finding, Khajavy et al. (2021) contended that a growth mindset was a strong predictor of student engagement. Additionally, and partially in accordance with the findings, Eren and Rakıcıoğlu-Söylemez (2020) reported a positive relationship between learners’ language mindsets and their engagement. The mediating role of FLE in the model is consistent with previous research that has shown that enjoyment is a significant predictor of engagement and achievement in foreign language learning (Dewaele and MacIntyre, 2016). The results suggest that fostering FLE may be an important factor in enhancing students’ engagement in English learning. Teachers can promote FLE by providing interesting and engaging materials, encouraging communication in the target language, and creating a positive and supportive classroom environment (Dewaele and MacIntyre, 2016).

Overall, the present study provides evidence for the importance of perceived teacher-student relationship, growth mindset, and FLE in promoting student engagement in foreign language learning. The results suggest that EFL teachers should focus not only on teaching language skills, but also on fostering positive relationships with students, promoting a growth mindset, and creating an enjoyable and engaging learning environment.

## Conclusion and implications

The aim of this study was to investigate how student engagement is predicted by factors such as perceived teacher-student relationships, growth mindset, and the mediating role of FLE. The findings of this study suggest that perceived teacher-student relationship, growth mindset, and foreign language enjoyment are important predictors of student engagement among Chinese English learners. The study found that perceived teacher-student relationship and FLE have a direct effect on student engagement, while growth mindset has an indirect effect on student engagement through the mediation of FLE. Overall, the research article highlights the importance of promoting positive teacher-student relationships, foreign language enjoyment, and a growth mindset as key predictors of student engagement in foreign language learning. Teachers can enhance student engagement and motivation in foreign language learning, ultimately improving student learning outcomes, by implementing effective strategies to support these factors.

The current study highlights the significance of the student-teacher relationship, since it encourages and motivates teachers to demonstrate new teaching strategies and evaluate their theories in order to build relationships with students that will increase motivation and lead to their success and engagement. Pupils’ behavior is strongly connected to their motivation, and teacher support via interpersonal relations is linked to pupils’ subject-related motivational aspects. Therefore, it is the responsibility of the educators to increase students’ motivation and motivate them to develop positive learning strategies (Yildirim, 2012). The more encouragement pupils receive from teachers, the more motivated they are to maintain language learning, which affects their ability to control their social-emotional behavior (McElhaney et al., 2008). It is important to note that teachers who have positive relationships with their students create a learning environment that addresses both the students’ emotional and academic needs. As official teaching begins, a good student-teacher relationship establishes the foundation for effective changes to be made to the learning environment for students. Students who feel their teacher doesn’t encourage them in the class are far less enthusiastic and engaged in the learning environment (Tyler and Boelter, 2008).

This study can also be significant for students since it enhances their understanding of the idea that educational methods are jointly constructed events and that teachers do not bear full responsibility for their success. In fact, students are dominant in offering a variety of ways to accomplish their goals. They attempt to establish a friendly relationship with their instructors, and this intimacy boosts their self-confidence and ambition. Students exhibit greater motivation and dedication in their performances when they are aware of their crucial role in the learning process. Overall, the outcomes of this study contribute to the literature on the importance of teacher-student relationship, growth mindset, and FLE in foreign language learning. The study suggests that these variables are interrelated and can have a significant impact on student engagement.

The results suggest that educators and policymakers should consider the importance of teacher-student relationship, growth mindset, and FLE in designing effective foreign language teaching strategies. EFL instructors should focus on boosting positive relationships with their students, which could be achieved through small gestures such as addressing students by their names, showing interest in their lives, and encouraging their participation in class discussions. Additionally, teachers could foster growth mindset by emphasizing the value of effort and persistence rather than innate ability, and by providing feedback that focuses on the process of learning rather than just the end result. Finally, enhancing FLE could be done by integrating enjoyable and engaging activities into the curriculum that align with students’ interests, preferences, and needs. Also, the findings underscore the role of collaboration between educators, parents, and students in promoting student engagement and success. Educators should communicate with parents about the significance of the student-teacher relationship, growth mindset, and FLE in foreign language learning, and encourage parents to support their children’s learning by providing a supportive and motivating environment at home. Students, on the other hand, could benefit from being educated on the importance of the student-teacher relationship, growth mindset, and FLE, and how they could contribute to their own learning process by actively engaging in class, seeking feedback, and reflecting on their learning progress.

Some limitations might affect the outcomes. One limitation of this study is the use of self-report measures, which are subject to biases and may not accurately reflect students’ actual experiences. Another limitation is that the study was conducted with a sample of Chinese English learners, so the transferability of the findings to other populations may be limited. Future studies should examine the generalizability of these findings to other populations and languages. Furthermore, it is important to acknowledge that cultural differences may influence the development of growth mindset in students. In this regard, previous research has shown that students from different cultural backgrounds may develop growth mindset in different ways (Yeager and Dweck, 2012). As such, future research could investigate how cultural factors may influence the development of growth mindset in EFL learners in different cultural contexts. This would provide a more comprehensive understanding of the constructs that contribute to the development of growth mindset in EFL learners and inform the development of effective interventions to promote student engagement and FLE. Also, this study was cross-sectional, so causal inferences cannot be made. Future research should use a longitudinal design to examine the causal relationships among perceived teacher-student relationship, growth mindset, FLE, and student engagement in foreign language learning. Finally, future studies should explore the role of other variables, such as motivation, self-efficacy, and anxiety, in the relationship among these variables.

## Data availability statement

The data analyzed in this study is subject to the following licenses/restrictions: The raw data supporting the conclusions of this article will be made available by the authors, without undue reservation. Requests to access these datasets should be directed to HL, lihaoting422@126.com.

## Ethics statement

The studies involving human participants were reviewed and approved by the Marxist Institute, Ningbo University, Ningbo, China. The patients/participants provided their written informed consent to participate in this study.

## Author contributions

The author confirms being the sole contributor of this work and has approved it for publication.
